# Redefining quality in cell and gene therapies: Lessons from implementing electronic QMS in academic cGMP facility

**DOI:** 10.1016/j.ymthe.2025.03.050

**Published:** 2025-03-31

**Authors:** Xia Wu, Amaia Cadinanos-Garai, Vivian Quach, Eric Jurado, Alix Vaissié, Mohamed Abou-el-Enein

**Affiliations:** 1 USC/CHLA Cell Therapy Program, University of Southern California and Children’s Hospital of Los Angeles, Los Angeles, CA 90033, USA; 2 Division of Medical Oncology, Norris Comprehensive Cancer Center, Keck School of Medicine, University of Southern California, Los Angeles, CA 90033, USA; 3 Department of Stem Cell Biology and Regenerative Medicine, Keck School of Medicine, University of Southern California, Los Angeles, CA 90033, USA; 4 Department of Regulatory and Quality Sciences, Alfred E. Mann School of Pharmacy and Pharmaceutical Sciences, University of Southern California, Los Angeles, CA 90033, USA

**Keywords:** cell and gene therapy, academic current Good Manufacturing Practice facilities, electronic quality management systems, manufacturing challenges, regulatory compliance

## Abstract

Manufacturing cell and gene therapies (CGTs) involves complex processes that require robust quality management, especially within academic current Good Manufacturing Practice (cGMP) facilities, where resources are often limited. Traditional paper-based quality management systems (QMSs), while initially convenient, often become burdensome, leading to errors, poor traceability, and compliance risks. Electronic QMSs (eQMSs) are gaining recognition for their ability to centralize and automate key quality processes, significantly enhancing operational efficiency and regulatory readiness. Through an in-depth case study of the University of Southern California and Children’s Hospital of Los Angeles academic cGMP facility, this review demonstrates tangible improvements achieved by adopting an eQMS. Practical insights gained from this experience are shared, including careful selection of eQMS platforms, phased rollout strategies, and comprehensive staff training. The review also addresses common implementation challenges and suggests practical solutions to overcome them. Lessons learned and strategies discussed here can serve as valuable guidance for other academic institutions considering eQMS adoption. Ultimately, embracing an eQMS enables academic CGT manufacturers to operate more efficiently and stay ahead in a fast-moving field.

## Graphical abstract

**Figure:**
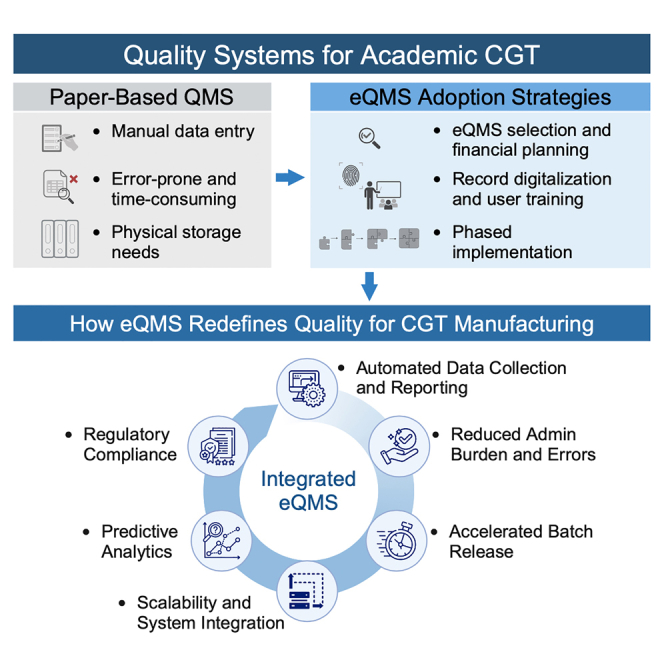

## Teaser

Academic cGMP facilities play a critical role in CGT innovation but often operate with limited resources. This review demonstrates how implementing an eQMS enhances operational efficiency and compliance, redefining quality management in advanced therapeutics. A case study offers a practical roadmap for adoption in academic settings.

## Introduction

Cell and gene therapies (CGTs) are advanced therapeutic modalities that utilize living cells, genetic engineering, and tissue engineering to treat diseases that lack effective interventions.^1^^,^^2^^,^^3^^,^^4^^,^^5^ Over the past two decades, several CGTs have entered the market; however, many early approved products struggled to achieve sustainable commercial success, partly due to high manufacturing costs and challenges with reimbursement.^6^^,^^7^ The emergence of chimeric antigen receptor-T (CAR-T) cell therapies has provided a more viable commercial model, driven by clinical efficacy and a maturing manufacturing infrastructure.^8^ Nevertheless, the development of CGT products is inherently complex and costly owing to the use of biological material, which introduces variability, complicates consistent manufacturing, and increases regulatory requirements.^1^^,^^9^^,^^10^ Autologous therapies, in particular, require individualized manufacturing workflows tailored to each patient, whereas allogeneic products often require additional genetic engineering to reduce the risk of immune rejection.^11^ Moreover, because terminal sterilization is not feasible, stringent aseptic processing is mandatory to ensure product safety.^12^ Finally, specialized cryopreservation and cold chain logistics are essential to preserve cell viability throughout storage and delivery.^13^

An effective quality management system (QMS) is thus essential to ensure product consistency, patient safety, and compliance with evolving regulations.^14^ Traditionally, many small-scale or low-resource manufacturers, such as academic current Good Manufacturing Practice (cGMP) facilities, relied on paper-based QMS approaches. Although these systems can be relatively straightforward to implement, they face significant drawbacks, including manual data entry errors, extensive physical storage needs, and inherent challenges in auditing and traceability. Managing documentation across multiple teams also becomes cumbersome, increasing the likelihood of deviations and compliance lapses. By contrast, electronic QMSs (eQMS) have emerged as a promising alternative, even for academic facilities, provided they can accommodate the initial investment. An eQMS centralizes critical processes on a digital platform, enabling real-time monitoring, automated version control, and secure data backup and recovery while ensuring compliance through continuous oversight and traceability.^15^ Although adopting an eQMS entails upfront costs and a learning curve, its long-term advantages make it a strategic choice for sustaining CGT manufacturing operations.

In this review, we first outline the fundamental principles of QMS in CGT manufacturing. We then examine the advantages and challenges of transitioning from paper-based systems to an eQMS, particularly in resource-limited academic cGMP environments. To the best of our knowledge, this is the first comprehensive review of eQMS adoption specifically for cell therapy manufacturing. Key considerations for eQMS selection, implementation, and optimization are discussed, followed by a case study illustrating real-world application. By sharing lessons learned from this process, we provide a practical guide for academic facilities seeking to enhance quality management in CGT manufacturing through digital transformation.

## Unique challenges in CGT manufacturing and academic cGMP facilities

Regulatory requirements for CGT products impose rigorous demands on facility design, process control, environmental monitoring (EM), and documentation, which can be particularly challenging for academic cGMP programs.^7^^,^^16^^,^^17^^,^^18^ The individualized nature of many CGT products adds significant operational complexity, as investigators often target diseases affecting high-risk patient groups and rare disease populations.^19^ Although the fundamental cGMP principles remain unchanged, the rapid transition from bench research to early-phase clinical manufacturing can outpace the ability of academic programs to establish and maintain standardized quality systems.^20^ Moreover, regulatory authorities require manufacturers to demonstrate comparability when making changes to cell therapy manufacturing processes. This includes modifications such as changes in critical equipment, raw materials, or process parameters, which must be justified through risk-based assessments to ensure that product safety and efficacy remain uncompromised. ^21^ In an environment where science and technology evolve rapidly, ensuring every experiment aligns with cGMP requirements can become an immense administrative task for academic facilities.

Workforce shortages add to the operational challenges faced by academic cGMP facilities.^18^ High turnover rates among postdoctoral researchers, graduate students, and junior technicians create ongoing disruptions, as they frequently transition to other positions. Since CGT manufacturing requires specialized training in aseptic processing, equipment operation, and product development, frequent onboarding and knowledge transfer become chronic issues. Quality assurance (QA) and quality control (QC) roles are typically understaffed, forcing personnel to juggle a wide range of responsibilities such as deviations, SOP revisions, and project-specific assay development and qualification. Concurrently, routine yet time-intensive cGMP tasks such as EM and equipment maintenance and qualification further strain available bandwidth, leaving little capacity for process optimization and analytical development tasks. More broadly, ensuring compliance with cGMP regulatory requirements increases administrative overhead and maintenance costs, outpacing available resources in academic settings.

Academic cGMP facilities often rely on short-term grants, limited institutional support, or philanthropic funding that may be insufficient for long-term infrastructure maintenance and expansions.^12^ Unlike industrial manufacturers that allocate substantial budgets for automation, digital infrastructure, and dedicated QMSs, academic institutions must balance their resources across multiple research projects. This fragmented focus can hinder the adoption of state-of-the-art manufacturing technologies.^22^ Furthermore, advanced analytics platforms for real-time monitoring and predictive modeling,^23^ commonly used by larger manufacturers, are often inaccessible to academic teams, limiting their ability to manage process deviations proactively and exacerbating compliance risks. Hence, sustainable operation in these resource-limited settings hinges on a robust, well-designed QMS that ensures compliance and data integrity while optimizing resource allocation and streamlining personnel responsibilities.

## Fundamentals of QMSs for CGTs

CGT manufacturers must meet multiple overlapping regulatory standards to ensure product quality and efficacy. The U.S. Food and Drug Administration (FDA) mandates stringent oversight of raw materials, in-process monitoring, and product release testing. In parallel, ISO 9001 and ISO 13485 outline system-wide principles for quality management and device quality. The ICH guidelines (Q2, Q6, Q7, Q9, and Q10) outline best practices and risk-based strategies relevant to CGT manufacturing. ICH Q2(R2) focuses on the validation of analytical procedures and ICH Q6 addresses test criteria and specification for drug substances and products. ICH Q7 defines GMP standards for active pharmaceutical ingredients, which can be adapted for CGT intermediates such as vectors or cell banks. ICH Q9 establishes risk management principles, incorporating methodologies like failure modes and effects analysis and fault tree analysis. ICH Q10 provides a comprehensive QMS framework. Additionally, all products should comply with the Code of Federal Regulations (CFR) section 21 CFR Parts 210 and 211 for drug products, and 21 CFR Part 1271 for human cells, tissues, and cellular and tissue-based products. These standards converge to demand a robust QMS that addresses the complexities of handling living materials under aseptic conditions. Table 1 outlines the key QMS elements for CGT operations along with scenarios of practical challenges in CGT workflows.

**Table 1:** Key QMS elements for CGT manufacturing

QMS element	Definition and purpose	CGT-specific requirements and challenges	Example scenario	Potential Pitfalls	Recommended best practices
1. Document control and management	establishes a system for creating, revising, distributing, and archiving SOPs, batch records, and other controlled docs	• frequent protocol updates require strict version control • complex multi-step procedures demand detailed instructions	a T cell engineering SOP is updated with new transduction parameters	• using obsolete SOPs can affect product viability or potency • storing SOPs in multiple uncoordinated locations	• use validated document management tools with version control • assign final approval to QA • grant users only the necessary privileges
2. Training management	ensures personnel have documented competencies for specialized tasks (aseptic techniques, vector handling, cryopreservation)	• rapidly evolving CGT methods demand continuous retraining • advanced skills required beyond standard cGMP knowledge	a new operator needs to learn sterile fill-finish for CAR-T products	• high turnover can erode expertise if training is not repeated and recorded • untrained personnel performing incorrect procedures	• map individual staff skills • perform routine training re-qualifications (e.g., annually) • link training to SOP revisions
3. Supplier and vendor management	defines processes for selecting, qualifying, and monitoring external providers of materials or services	• raw materials (e.g., cell culture media, cytokines) can impact final product functionality • lot consistency is essential	a facility sourcing critical reagents from an external vendor must ensure no changes in production or lot-to-lot variability	• relying on a single supplier for critical reagents risks supply disruptions • delayed responses to supplier deviations	• perform risk-based supplier qualification, audits, and periodic reviews • use quality agreements with change notification
4. Material management	tracks and controls all incoming materials (cells, media, reagents) from receipt to release or rejection	• high variability in input materials demands robust traceability • strict storage conditions are required • complex procedures need careful supply planning	donor leukapheresis arrives for autologous T cell therapy production they must be labeled, quarantined, and tested before release	• temperature excursions or improper handling can reduce cell viability • paper-based logs increase risk of mix-ups • inaccurate inventory can disrupt production schedule	• maintain inventory databases with lot-to-product traceability • monitor freezers and incubators with validated systems
5. Non-conformance and deviation management	establishes procedures to identify, document, investigate, and resolve deviations from approved methods	• small CGT batches amplify minor errors, making cross-functional root cause analysis essential	a sterile filter clogged during media addition in a CAR-T run, prompting investigation into whether the cause was the filter or operator technique	• delayed or isolated investigations lead to repeated errors and ineffective root cause analysis	• standardize root cause analysis forms capturing technical, procedural, and human factors • cross-functional review (QA, QC, MFG)
6. Change management	controls modifications to materials, methods, or documentation through formal review and approval	• CGT processes may evolve quickly, and even minor changes can impact CQAs	switching to serum-free media for T cell expansion requires pilot runs, comparability studies, and updated SOPs	• poorly documented changes and informal, unrecorded discussions increase the risk of non-compliance	• justify changes based on CQA impact and time them appropriately for regulatory submissions
7. Risk management	uses FMEA or fault tree analysis to identify and mitigate risks to product quality	• CGT introduces unique risks such as off-target genome editing	• use of CRISPR requires off-target analysis • FMEA can identify critical steps such as guide RNA design and Cas nuclease fidelity	• outdated risk assessments and underestimating severity	• integrate risk analysis early in process design and update continuously
8. Audit management	conducts internal and external audits to confirm QMS compliance and identify gaps	• CGT inspections require traceability, comprehensive data logs, and chain-of-identity for patient-specific lots	during FDA inspection, quick retrieval of deviation logs and CAPA outcomes from the past 12 months is essential	• unaddressed audit findings and incomplete records lead to repeat non-compliances and poor inspection readiness	• maintain a risk-based audit plan focused on high-risk areas, with centralized findings linked to CAPAs for accountability

CAPA, corrective and preventive action; CAR, chimeric antigen receptor; CQA, critical quality attribute; FMEA, failure modes and effects analysis; MFG, manufacturing; SOP, standard operating procedure.

Early cGMP suites established in universities and research hospitals often began as extensions of cell-processing labs and transfusion medicine units, gradually integrating cGMP principles.^24^ Quality systems were initially built around written SOPs, batch records, and training manuals, stored in physical binders or filing cabinets. This paper-based model was straightforward to implement and, for facilities producing limited batches in investigator-initiated trials, provided sufficient traceability for local regulatory compliance. As CGT technologies advanced and product variability increased, the growing need for specialized standard operating procedures (SOPs) intensified documentation burdens, making version control, record retrieval, and compliance particularly challenging for academic cGMP units scaling their clinical production.^10^^,^^25^ The expansion of CGT pipelines and increased industry involvement further intensified operational demands. This evolution exposed the need for more robust, digitalized systems that can maintain real-time auditability and support continuous process improvements.^26^

## Opportunities in transitioning to eQMS

An eQMS is a validated software platform that centralizes and automates key quality processes,^27^ such as document control, deviation and corrective and preventive action (CAPA) management, change control, training, audit oversight, material management, and supplier qualification. Adoption of eQMS systems gained momentum in the mid-to-late 1990s with advancements in software development, networked computers, and enterprise platforms. Regulatory mandates such as FDA 21 CFR Part 11 and EU Annex 11, introduced in the late 1980s and 1990s to govern electronic records and signature management, further accelerated their implementation in the 2000s, particularly in life sciences and other highly regulated industries. Today, various specialized eQMS platforms are widely used, offering substantial technical and logistical benefits for CGT manufacturing (Table 2). In line with Industry 4.0’s emphasis on integrated, data-driven operations, eQMS solutions now replace many paper-based systems.^28^ The benefits of transitioning to an eQMS can be categorized into four main areas.

(1) Reduces administrative burden and errors: The transition from a paper-based QMS to an eQMS can markedly streamline the administrative workload in CGT manufacturing. Manual document handling in paper-based systems is inefficient, error prone, and increasingly unsustainable as CGT operations scale. An eQMS mitigates these challenges by automating version control, securing records with electronic signatures, and centralizing documentation. Such system prevents the inadvertent use of outdated protocols, minimizes redundancy in data entry, and enables real-time monitoring of activates. Several large-scale pharmaceutical and stem cell processing facilities have documented significant time savings, ranging from 16 weeks per year to nearly 60% faster throughput, when replacing paper-based recordkeeping with electronic systems.^26^ For small-batch products, reduction in paperwork can significantly shorten batch release timelines.
(2) Facilitates compliance with regulatory requirements: When preparing investigational new drug (IND) submissions, particularly the chemistry, manufacturing, and controls section, much of the required information comes from routine manufacturing records.^29^ A well-configured eQMS securely retains records with embedded audit trails for quick retrieval and verification. As regulatory submissions increasingly rely on standardized electronic formats like the electronic common technical document,^29^ eQMS can help to eliminate the need for manual scanning of voluminous paper records. It also simplifies audit preparation by providing instant access to records, avoiding the cumbersome retrieval and reassembly of paper files, which are prone to misplacement. Additionally, an eQMS enables review by exception, allowing auditors to quickly identify non-conformances without sifting through extensive paper records. Studies show that organizations transitioning from paper-based systems to an eQMS can reduce the time spent on audit preparation, execution, and reporting by up to 70%.^30^^,^^31^
(3) Strengthens traceability in CGT manufacturing: In CGT manufacturing, the use of diverse donor materials and the need for cryopreservation for offsite shipment require precise records to ensure chain of identity and traceability.^32^^,^^33^ An eQMS addresses these needs through controlled access rights, encryption, and real-time data tracking. A retrospective analysis at a health care-based cell therapy lab reported 15 off-site manufacturing protocols supported between 2012 and 2022, with 13 active in 2022 and 76 product infusions that year.^34^ This range of protocols demonstrates the need for robust tracking, which an eQMS ensures through full traceability and unbroken audit trails, providing a level of oversight that paper-based systems cannot match. Reliable traceability is of particular importance in investigating deviations, evaluating root causes, or satisfying regulatory queries.
(4) Offers advanced capabilities such as predictive analytics: Some eQMS platforms now incorporate predictive analytics to identify emerging trends in manufacturing data.^35^^,^^36^^,^^37^ By integrating historical metrics on personnel activity, EM, and product parameters, these systems can detect early signals of deviations before they compromise product quality. This enables proactive interventions, such as adjusting processing conditions or retraining personnel. Predictive analytics can aid in resource allocation and process optimization, for example, by analyzing equipment usage to schedule preventive maintenance and avoid downtime.^38^ Similarly, trend analyses of environmental data can identify contamination risks, prompting targeted improvements in cleaning procedures or operator flow patterns. Predictive analytics can also strengthen CGT supply chain resilience by optimizing inventory, forecasting demand, and mitigating disruptions.^39^

**Table 2:** Comparison of Paper-Based QMS and eQMS

Aspect	Traditional paper-based QMS	eQMS
**Operational efficiency**		

Data entry	manual and time-consuming; prone to transcription errors	automated and faster, with built-in validation and audit trail capabilities
Human errors	higher risk of misfiling, illegibility, and incomplete entries	reduced error rates due to digital prompts and controlled fields
Document search and retrieval	slower, manual lookup that can be labor-intensive, and typically limited to individuals familiar with record locations	instant search functionality with version control in a centralized database
Reporting	manual compilation of data; real-time or large-scale reporting is generally unfeasible	automated reporting with real-time dashboards and rapid metric generation
Version control and updates	requires collection and destruction of outdated copies; increased risk of incomplete distribution	instant rollout of new or updated documents across all users
Collaboration	requires physical presence or mailing copies for feedback and approval	enables simultaneous access and review from multiple locations
Storage requirements	requires extensive physical storage space that increases over time	digital/cloud-based storage; eliminates the need for physical archives

**Compliance and regulatory requirements**		

Validation and regulatory scrutiny	no software validation required, but must meet GMP standards	requires software validation (e.g., 21 CFR Part 11) and secure access controls
Compliance	managed through manual logs and sign-offs; greater risk of missed updates or delayed reporting	automated tracking of cGMP standards, with built-in alerts for deviations and training needs
Audit readiness	record retrieval is time-consuming; increased risk of misplaced documents	rapid retrieval of electronic records for internal and external audits

**Risk and data security**		

Data security	susceptible to physical damage (e.g., fire, flood), unauthorized access, or theft	protected by encryption, tiered user access, and reliable backup protocols
Cybersecurity risks	immune to cyber threats but vulnerable to physical loss, tampering, and uncontrolled or unsecured distribution	requires cybersecurity measures in place, including intrusion detection and vulnerability scanning
Disaster recovery	difficult or impossible to recover from severe physical damage	systematic backups and recovery procedures enable quick restoration after disruptions

**Onboarding and maintenance**		

Onboarding process	familiar workflow with minimal initial training required	requires training in software use, system administration, and validation procedures
Initial Cost	low upfront cost; no specialized IT infrastructure needed	high initial investment for software, hardware, implementation, and validation
Long-term costs	accumulated costs from manual labor, rework, and physical storage	lower ongoing costs due to reduced labor, storage, and compliance issues
Scalability	physical storage and document coordination become increasingly complex as scalability demands grow	easily scalable for higher data volumes, users, and process expansion; integrates with clinical and vendor systems
Maintenance	minimal IT support; routine upkeep of physical archives	requires regular IT support, software updates, and security reviews

## Challenges in implementing eQMS

Academic cGMP facilities often operate under a broader research mandate than commercial entities, manufacturing multiple types of CGTs across various clinical phases.^18^ This multiproduct environment requires flexible processes for early-phase trials and stringent QCs for later-stage trials.^40^ Balancing these diverging needs within a unified QMS is challenging, as academic cGMP sites often struggle to maintain a phase-appropriate system that is both scientifically rigorous and feasible to execute, particularly when product pipelines span diverse therapeutic modalities.

Implementing an eQMS in these settings faces multiple technical and integration hurdles. Many platforms, originally designed for corporate biopharmaceutical sectors, are less adaptable to the lean staffing models and modular workflows of academic cGMP facilities.^25^^,^^41^ Rather than using a single purpose-built platform, academic facilities often rely on a hybrid ecosystem of laboratory information management systems (LIMS), shared drive databases and spreadsheets to manage quality documentation.^42^^,^^43^^,^^44^ Although this approach may provide short-term flexibility, it can fragment data flows and complicate consistency. Moreover, centers with QMSs for blood banks or stem cell transplantation,^45^^,^^46^ may attempt to extend their systems to cell therapy manufacturing. However, these systems are often rigid, lacking flexibility for modifications or tailoring, and fail to provide specialized functionalities required by CGT manufacturing.

A current limitation of eQMS in academic cGMP facilities is the lack of seamless integration with equipment monitoring systems. Academic labs often rely on instruments from multiple vendors with incompatible data formats and communication protocols. These platforms do not provide an application programming interface (API) for direct data transfer, which would enable an eQMS to automatically retrieve and process data in real time. Without an API, equipment data must either be manually uploaded by personnel, which is labor intensive and prone to errors, or transferred using custom scripting, where information technology (IT) teams program automated workflows to extract and transfer data. However, this method is unreliable, as software or firmware updates can break the script, leading to data gaps and misaligned timestamps. To effectively address this challenge and integrate equipment monitoring data into the eQMS, custom solutions and collaboration between different vendors will be required.

Budgetary constraints and resource limitations further complicate eQMS adoption. A survey of Canadian GMP cell therapy centers found that limited funding restricts investment in infrastructure, regulatory compliance, and QC, with many centers struggling to sustain GMP compliance and scale manufacturing.^47^ This financial structure not only reduces the ability to purchase eQMS solutions but also impedes hiring or training the dedicated personnel required to configure and maintain such systems.^18^ Costs can escalate quickly to cover software licensing, customization, data migration, and ongoing technical support. For instance, City of Hope reported an estimated $149,000 one-year implementation cost for Labware LIMS, excluding operating expenses.^42^ Beyond finances, employee resistance to shifting from paper-based workflows and the time needed to learn new systems can delay or derail implementation.^48^ This combination of budget, multi-phase manufacturing needs, and human resource challenges makes eQMS adoption a protracted decision in academic CGT facilities.

Ensuring data integrity and security in academic cGMP facilities may pose another challenge due to limited resources and reliance on legacy IT systems. Additionally, interfacing with clinical databases or hospital electronic medical records to maintain traceability from donor to patient requires eQMS solutions that are also compliant with the Health Insurance Portability and Accountability Act. To meet these demands, eQMS must incorporate robust audit trails, compliant electronic signatures, access control mechanisms, and safeguards like multifactor authentication and role-based permissions. Data entries must be time stamped, user attributed, and protected from deletion or unauthorized modification. Any inconsistencies in audit trails or version histories can trigger regulatory concerns. Staff turnover further complicates matters, potentially leaving eQMS systems under-monitored or misconfigured. To mitigate this, academic facilities must establish and rigorously follow SOPs for system maintenance, data governance, and security. Ongoing collaboration with eQMS vendors is essential to ensure that training, access controls, and system validations continue seamlessly, even with personnel changes.^49^ Moreover, cybersecurity risks remain a constant threat, requiring eQMS vendors to provide regular updates to mitigate impact of vulnerabilities, identify security threats, and avoid unauthorized access.

## Strategies for successful eQMS adoption in academic cGMP facilities

Selecting an eQMS for an academic cGMP facility requires a criteria-based approach. The facility should first define key priorities, such as out-of-the-box functionality, user configurability, instrument/data interface capabilities, cost structure, and long-term vendor support, and then evaluate how each solution addresses those needs. For example, City of Hope compared four candidate platforms using pre-established metrics (e.g., batch record flexibility, custom reporting features).^42^ The highest-scoring system provided integrated modules for document control, freezer inventory, and deviation management. This data-driven method helps academic sites to avoid eQMS platforms lacking essential CGT features or requiring excessive customization.

Careful financial planning is critical, as initial eQMS expenses, including software licenses, validation, hardware upgrades, and staff training, can be substantial. Ongoing costs may include annual support fees, updates, or pay-as-you-go charges that scale with data volume or the number of concurrent users. Although initial eQMS implementation costs may seem daunting,^42^ facilities often achieve mid- to long-term savings by reducing manual labor, speeding deviation resolution, and minimizing compliance-related disruptions. Some centers secure institutional support or targeted grants to offset startup costs and ensure sufficient resources for ongoing training and maintenance. For instance, the California Institute for Regenerative Medicine (CIRM) has established a unique funding mechanism for cGMP facilities in California, aimed at supporting QMS implementation and enhancements for CGT manufacturing—a model not widely available elsewhere.

A phased implementation strategy helps academic facilities to manage the complexities of eQMS adoption across multiple therapeutic programs. Rather than launching every module at once, many facilities start with critical areas, such as document control, inventory, or training management, where an immediate impact is most likely. By mapping paper-based workflows and introducing targeted eQMS functionalities, teams can assess system performance on a smaller scale. This trial run identifies configuration issues, refines usability, and measures staff acceptance. Demonstrating improvements, such as quicker SOP updates or faster CAPA actions, can boost productivity and encourage broader adoption. Each phase should include a feedback mechanism to address concerns and optimize the system before full deployment.

Effectively managing data migration, validation, and user adoption is critical for successful eQMS implementation. Paper-based records (e.g., SOPs, CAPA logs, training documents) must be digitized with consistent naming conventions and audit trails, while outdated materials are removed. Rigorous software validation, aligned with Title 21 CFR Part 11, FDA 21 CFR Part 820, ISO 9001:2015, and CGT-specific regulations, ensures critical features (access controls, electronic signatures) function properly. Ongoing role-specific training and clear communication build staff confidence, reinforce best practices, and minimize errors. Finally, periodic QA audits help to identify process inefficiencies and non-compliance issues, ensuring continuous system optimization. Table 3 summarizes the key criteria and lessons learned from our experience using eQMS (Bluecord), providing guidance for academic cGMP facilities in selecting eQMS solutions that meet their specific needs.

**Table 3:** Key selection criteria and lessons learned in eQMS implementation

Criterion	Description	Lessons learned
1. Configurability for CGT workflows	the eQMS must support diverse CGT manufacturing protocols, allow workflow customization, and scale from process development to clinical trials	while pre-built templates provided a starting point, significant modifications were necessary to align with our workflows a system with flexible customization was essential
2. Document control and deviation management	a centralized system for SOPs, batch records, CAPAs, and audits ensures version control, notifications, and controlled access	the eQMS improved compliance and documentation, but consistent QA oversight and staff training were key to ensuring deviations and CAPAs were properly managed
3. Total cost of operation	costs include validation, maintenance, training, and customizations some systems operate on licensing fees, while others use an upfront and service-based pricing model	the Bluecord model was more academic-friendly, operating on upfront fees plus annual service costs instead of recurring licensing fees and limited number of users, simplifying long-term budgeting and planning
4. Integration with laboratory monitoring systems	many lab monitoring systems lack APIs, making automated data transfer into the eQMS difficult options include manual data downloads or custom scripts, which can be unreliable	connecting equipment monitoring systems was more complex than expected as a workaround, we relied on manual data extraction, but a standardized integration solution remains an open prospect
5. Interoperability with LIMS	integrating eQMS with LIMS improves experimental data management and supports seamless transition to GMP manufacturing.	Bluecord developed a module to help digitalize process and analytical development experiments, improving traceability and data organization
6. Vendor support and system updates	a vendor’s ability to provide feature updates, security patches, and regulatory support is critical for long-term system viability	regular system updates and direct vendor feedback accelerated development of features tailored to our needs
7. Data security and integrity	robust audit trails, encryption, and controlled access are essential for regulatory compliance (21 CFR Part 11)	university IT approval for the eQMS took longer than expected due to unfamiliarity with the system extensive discussions were required to address data access, encryption, and security concerns
8. Scalability and phase-appropriate controls	the eQMS must scale from process development to clinical production across all trial phases, without requiring disruptive system migrations	we developed a phase-appropriate electronic batch record and lab module for process development in alignment with other academic cGMP sites
9. User training and change management	academic settings require accessible training modules and clear change tracking to accommodate high turnover and evolving protocols	we implemented a user-friendly training interface that automatically alerts staff of required updates and ensures alignment with QA training schedule
10. Long-term sustainability and IT governance	regular system maintenance, security updates, and IT oversight are necessary for long-term functionality	ongoing system maintenance and revalidation required close collaboration with Bluecord to ensure continued compliance and system performance

## Case study: Transformative impact of eQMS in an academic CGT facility

The University of Southern California and Children’s Hospital of Los Angeles (USC/CHLA) academic cGMP facility is the result of a collaborative investment between the Keck School of Medicine, the Keck Medical Center of USC, and CHLA. Completed in late 2022 and officially opened in 2023, the 3,184-square-foot facility includes 6 production suites and specialized labs for QC, vector engineering, and process development. The facility’s mission is to drive translational research in CGTs by offering manufacturing services and in-house release testing for both internal investigators and external partners. From the outset, the facility team recognized that paper-based quality approaches would struggle to keep up with the evolving regulatory and scientific demands of CGT. After surveying multiple vendors, the team selected Bluecord, a platform specifically designed for CGT operations. Bluecord’s modular structure and user-friendly interface were ideal for adapting to the facility’s growing product lines. Bluecord worked closely with the USC/CHLA team to customize the system to meet the unique needs of a newly established academic cGMP setting, avoiding rigid, preconfigured workflows. Notably, the cost of implementing and servicing Bluecord represented only 5% of the facility’s yearly operational budget (excluding personnel costs), making it a highly cost-effective solution.

In October 2022, we began the initial phase of eQMS implementation by rolling out the first three modules—document control, training, and inventory. Bluecord provided both remote and on-site training for QA and key cGMP team members. To ease the transition, Bluecord offered a sandbox platform, Bluecrawl, allowing users to familiarize themselves with the system before its official launch. As a newly established facility, only 1 year’s worth of inventory records required digitization. Since we had no paper-based training records, SOPs, or deviation/CAPA records at the time, no data migration was necessary. System setup and refinement were supported through periodic meetings with the Bluecord team to address functionality improvements and optimize workflows.

**Figure 1:**
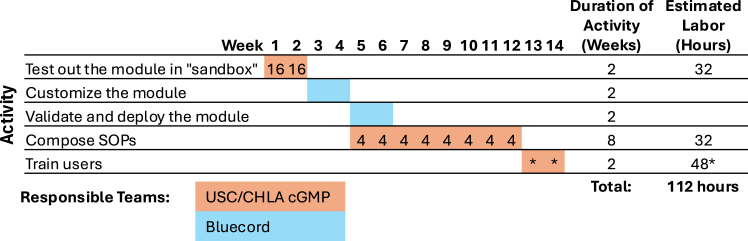
Workflow for Bluecord eQMS module implementation Typical workflow for implementing a Bluecord eQMS module at the USC/CHLA cGMP facility, along with the estimated labor (in hours) associated with each activity. The responsible teams are color-coded: orange for the USC/CHLA cGMP team and light blue for Bluecord team. Note that the labor incurred by Bluecord team is not included in the calculation. ∗The labor hours for training are based on a team of 12 trainees, but actual training time may vary depending on team size and trainee roles.

As the QMS developed, additional modules were gradually integrated. Figure 1 illustrates a typical workflow for implementing a new Bluecord eQMS module. The process begins with cGMP team members testing the module in the sandbox and providing feedback along with user requirement specifications to Bluecord. The Bluecord team customizes the module, performs validation, and provides reports for QA review and approval. Once validated, the module is deployed on the platform. During this process, the team drafts relevant SOPs and controlled documents, undergoes training, and integrates the module into daily operations. The typical implementation cycle spans an average of 14 weeks per module, with an estimated effort of 112 labor hours required from the facility team. By 2024, we had implemented 22 Bluecord eQMS modules, covering QA tasks (audits, CAPA, change control, training, and vendor management), manufacturing processes (electronic batch records, production logs), QC activities (testing, EM, stability, and lab notebook), and a comprehensive inventory system for tracking formulations, supplies, equipment, and cryo inventory. The following scenarios demonstrate how specific modules, such as inventory management and EM, improved daily operations.

(1) Scenario 1: Inventory module

Before implementing Bluecord, the facility used paper forms and hand-written stickers to track the life cycle of incoming materials, such as reagents and consumables. Operators manually recorded lot numbers, expiration dates, and brief product details on a form, while a separate sticker attached to each item provided only limited information. This system lacked a search function, requiring personnel to manually scan binders or rely on memory of storage room layouts to locate materials. Once an item was used, tracking its movement or consumption was cumbersome, often requiring cross-referencing multiple folders for certificates and release forms. This approach made record retrieval inefficient and traceability more complex.

**Figure 2:**
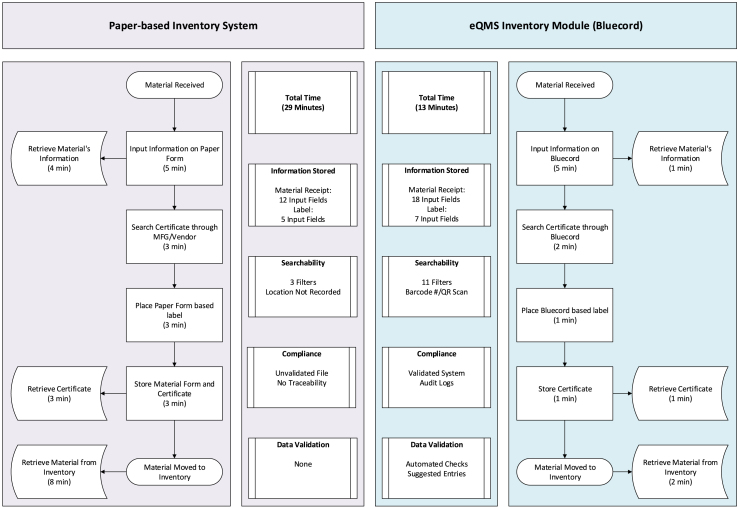
Inventory workflow using paper-based method vs. Bluecord eQMS The steps involved in two inventory workflows: paper-based vs. Bluecord eQMS. It outlines all processes related to inventory management, from receiving materials to retrieving items from inventory, and provides the estimated time associated with each step.

With Bluecord’s inventory module, all key metadata such as lot number, expiration date, and storage location and conditions are electronically captured. Built-in validation checks, including alerts for mismatched lot numbers or expired materials help to minimize transcription errors at the point of entry. Upon receipt, users can upload certificates of analysis directly, allowing the system to extract relevant fields and automatically populate the digital record, with operators verifying entries for accuracy. Each item is assigned a unique barcode which enables the operators to scan and update location changes or usage events in real time. A comprehensive audit trail logs every modification that links it to individual user credentials for full traceability. The system also includes a receipt function that logs key details upon material arrival, such as the date received, visual inspection results, and classification (e.g., critical or non-critical). These integrated features create a closed-loop inventory management system that covers the entire life cycle from initial receipt to final disposal. As a result, staff can quickly determine material status and location, retrieve associated certificates, and resolve batch discrepancies without manually searching through paper records. Figure 2 illustrates the efficiency gains of transitioning from a paper-based inventory system to Bluecord’s eQMS.

(2) Scenario 2: EM

The USC/CHLA facility implemented Bluecord’s EM module to enhance the tracking of environmental conditions in ISO-classified production suites. The system streamlines EM workflows by centralizing non-viable particle count, surface microbial contamination, and active air sampling data. EM templates, tailored to room layout and ISO requirements, guide operators during in-room monitoring to ensure complete sampling and documentation. By assigning predefined action thresholds to each sampling modality and linking them to room classifications and locations, operators can monitor results in real time. Any out-of-specification (OOS) event, such as elevated microbial counts or particle excursions, automatically triggers an alert to the QC manager, enabling rapid investigation and corrective action.

The EM module also tracks plate incubation with automated logging of start times, temperature switches, and incubation endpoints. QC operators can record colony counts, upload images for morphological assessment, and document species identification directly in the system. The platform also automates supervisory review and batch release decisions, issuing prompts once incubation and microbial identification are complete. By integrating these processes into a single interface, the system accelerates reporting, reduces transcription errors, and supports continuous monitoring of contamination trends across suites. Management can then proactively implement risk-based interventions, such as modifying disinfectant protocols or adjusting personnel flow, before transient contamination risks lead to production disruptions. Table 4 outlines the key features of the EM module, its implementation considerations, and its impact on operations.

**Table 4:** Key features of Bluecord eQMS EM module

Feature	Technical features	Key advantages	Implementation considerations
Template generation	• operators create customizable EM templates mapping rooms, sampling sites, and sampling types • templates auto-generate fillable EM records, reducing entry errors	• prevents omission of sampling sites and reduces prep time • ensures standardized collection of data, even with frequent staff rotations	• define template ownership (QA/QC) to control updates • ensure the template aligns with the most recent SOP and accommodates special-case sampling (e.g., additional sites) without compromising core structure
Threshold setting and notifications	• users configure alarm limits for each EM modality (e.g., viable air, surface, personnel monitoring and non-viable particle counts) • system flags OOS results in real-time and supports custom thresholds by room class and sampling modality (dynamic vs. static)	• enables proactive corrections when contamination exceeds pre-defined limits • configurable to US and EU specifications, and adaptable to other jurisdictions	• maintain a procedure to adjust thresholds as the facility evolves and validate their application to ensure consistent performance • define clear escalation rules and frequency of notifications to avoid “alarm fatigue”
EM Data reporting (inside cGMP)	• multiple operators can simultaneously log data in real time • operators enter initials upon completion of each site to ensure traceability • changes to the data are automatically recorded in the audit trail	• minimizes paper use in controlled areas to reduce contamination risk • tracks operator progress to prevent missed sampling sites • enables near-real-time feedback on contamination events	• provide cleanroom-compatible digital devices with protective covers • define user roles to control data entry and sign-off • consider connectivity solutions (Wi-Fi or local device sync) to handle outages
Plate incubation tracking (QC)	• logs incubation parameters: start date/time, temperature transitions, final readout date • permits multiple incubation conditions (e.g., 30°C–35°C, 20°C–25°C) • generates alerts if plates are not read within predefined timeframes	• prevents unread or expired plates • enhances traceability by linking each plate to a batch, room, and operator • supports on-schedule plate reading with automated notifications	• configure temperature/condition setpoints per pharmacopeial or SOP requirements • validate automated reminders to ensure timely plate reading • ensure that staff understand corrective actions if plates exceed recommended hold times
EM data reporting (QC)	• QC personnel record colony counts and morphology digitally • supports plate image uploads for colony documentation and ID • assigns criticality levels to identified microorganisms based on a custom library (e.g., fungi, spore-formers, gram negative)	• eliminates manual transcription and reduces errors • accelerates morphological and species-based analyses for faster corrective actions • facilitates trend analysis of recurring contaminants or high-risk microbes	• establish validated criteria for species identification and criticality classification (e.g., adopting internal or reference libraries) • train QC staff to properly annotate images and link them to the correct EM record
Data review and audit trail	• requires supervisory or area manager sign-off once sampling entries are finalized • tracks edits (date/time, user, nature of change) to form an audit trail • Supports e-signatures under FDA 21 CFR Part 11 and EU Annex 11	• simplifies inspections with a clear timeline of data entries and modifications • eliminates ambiguity about user credentials or late entries	• enforce role-based access control so that only designated staff can approve or amend records • validate e-signature protocols and electronic record retention rules (including external backup)
Data trending	• aggregates historical EM data (viable counts, non-viable particle metrics, contamination events) by room, operator, or time frame • visual dashboards highlight contamination hot spots or recurring patterns • allows cross-correlation of data with cleaning and production schedules	• enables proactive improvements, such as targeted cleaning or operator retraining • helps pinpoint root causes of contamination by linking environmental events to specific processes, facility cleaning or equipment usage	• customize trending parameters (e.g., sampling modality, date range, location) to enable tailored facility performance analysis • validate the charting/statistical tools for accuracy, especially if migrating data from legacy systems

## Discussion and future directions

The adoption of eQMS in CGT manufacturing presents a transformative opportunity to address key operational challenges and enhance efficiency. However, a major hurdle lies in the limited market size for academic institutions, which often makes it less attractive for vendors to develop cost-effective, tailored solutions. Many academic cGMP facilities struggle to justify the high capital investment, licensing fees, and training costs typically associated with eQMS platforms designed for large-scale biopharma operations. Nevertheless, the growing regulatory requirements for data integrity and the rapid expansion of advanced therapies necessitate the adoption of eQMS systems for academic institutions to remain competitive and compliant. Therefore, it is essential to create eQMS solutions that are both cost effective and specifically designed to address the unique needs of academic cGMP settings. As demonstrated in the case study presented here, tailoring eQMS solutions to meet the specific operational requirements of academic cGMP facilities can significantly improve performance. Moreover, aligning these systems with both academic and commercial manufacturing requirements can facilitate the transition from early-phase to late-stage production.

An important development in this area is the growing adoption of Bluecord within California’s CIRM-funded cGMP network. A key initiative, led by the USC/CHLA team, is the creation of a community module in Bluecord that enables controlled sharing of template protocols, best practices, and standardized processes among institutions while maintaining the confidentiality of proprietary information. This system is designed to foster collaboration across multiple academic sites, laying the groundwork for multi-site manufacturing models and consistent quality benchmarks. The initiative has the potential to not only enhance process consistency and scalability but also reduce redundant efforts, and facilitate technology transfer, ultimately accelerating the development and manufacturing of CGT therapies across the network.

Looking ahead, next-generation eQMS tools could incorporate artificial intelligence (AI)-driven capabilities for early detection of trends and deviations, utilizing pattern-recognition algorithms. For example, machine learning models trained on historical QC and EM data can identify operator-based deviations well before any OOS reading occur, enabling immediate corrective actions to prevent batch loss. AI-driven eQMS enhancements could also address challenges like high inter-batch variability and real-time process monitoring. Bayesian optimization models can refine process parameter control by continuously learning from in-line analytics. Natural language processing algorithms can also parse free-text entries in electronic batch records, helping QA managers detect anomalies, mislabeling, or incomplete data entries.^50^ Table 5 presents examples of AI-driven solutions that can help academic CGT keep pace with the evolving CGT landscape.

**Table 5:** Advanced eQMS capabilities for next-generation academic CGT manufacturing

Advanced eQMS feature	Technical features	Value proposition for academic CGT	Implementation considerations
AI-driven predictive analytics	• machine learning models trained on historical QC, EM, and batch data • automated anomaly detection and trend analysis (e.g., time-series forecasting)	• identifies process deviations early for high-variability autologous products • facilitates proactive root cause investigations and CAPA actions	• requires significant data volume/quality for model training • necessitates specialized data science expertise and robust computational resources
Chain-of-identity dashboards	• real-time tracking from donor sample to final infusion batch • bi-directional linkage of patient info, manufacturing logs, and shipping details	• ensures accurate chain-of-custody, critical in autologous therapies • minimizes risk of mix-ups	• requires integration with clinical databases, shipping vendor systems, and inventory modules • requires centralized identification protocols (barcode/QR)
Automated parametric release	• inline sensors and process data logs feed directly into eQMS • system-generated pass/fail criteria based on set thresholds for release	• reduces manual QC bottlenecks by approving product lots immediately upon meeting critical specs • invaluable for short-shelf-life CGT products	• demands validated instrumentation and robust data integrity measures • high burden of real-time data integration and continuous monitoring
Smart e-signatures and biometrics	• Multifactor authentication: e.g., access badges and biometric scans • time-stamped cryptographic signatures per 21 CFR part 11	• strengthens data integrity by preventing credential sharing • enhances compliance with advanced security requirements	• requires IT security collaboration for biometric data storage • must ensure compliance with privacy and institutional policies (e.g., HIPAA, GDPR)
Real-time collaboration tools	• built-in chat, annotation features, and versioning for multi-user SOP editing • configurable user roles for parallel reviews and e-approvals	• accelerates document approvals across multiple departments (QA, QC, PD) • facilitates remote collaboration, key for multi-site academic networks	• requires careful access control to prevent unauthorized editing • potential version conflicts if collaboration features are not well-regulated
Closed-loop risk management	• integration with Deviation/CAPA modules and predictive analytics • dynamically updated FMEA or fault tree analysis based on real-world batch data	• continually refines risk assessment as new data arrives • guides resource allocation for high-risk steps	• demands rigorous linking of risk assessment outputs to actual process deviations • tools must be flexible to accommodate emerging CGT technologies
AI agents	• trained on workflows as specified in SOPs • interfaces with internal and external digital tools to perform defined tasks	• manages increased workloads without the need for additional staff • automates repetitive, error-prone tasks	• requires thorough SOPs and reliable data inputs to train and direct the agents • potential hallucinations if AI agents lack complete or accurate data

GDPR, general data protection regulation; HIPAA, Health Insurance Portability and Accountability Act.

## Conclusion

Adopting an eQMS in academic CGT manufacturing presents significant opportunities to streamline operations, enhance compliance, and accelerate product release. However, integrating these systems requires careful planning, resource allocation, and ongoing support, which can challenge already strained academic budgets. Despite these hurdles, the expanding CGT pipelines and evolving regulatory requirements make an eQMS an essential tool for maintaining high manufacturing standards and advancing translational research. By embracing digital workflows, harnessing advanced analytics, and fostering a culture of continuous improvement, academic cGMP facilities can maximize the safety and efficacy of their therapies while navigating the evolving landscape of CGT innovation.

## Acknowledgments

M.A. is supported in part by the 10.13039/100000054National Cancer Institute under award number P30CA014089. The content is solely the responsibility of the authors and does not necessarily reflect the official views of the National Cancer Institute or the National Institutes of Health. The CIRM Manufacturing Network Grant (INFR5-14667) supported continued development of the USC/CHLA cGMP eQMS. The authors also acknowledge the valuable feedback and support from academic cGMP facilities, particularly Seattle Children’s, 10.13039/100005492Stanford University, 10.13039/100008476University of California, Irvine, and the 10.13039/100007707University of California, Davis.

## Author contributions

M.A. conceptualized the review. All authors contributed to the drafting of the manuscript, including the preparation of figures and tables. All authors critically reviewed and revised the manuscript for important intellectual content. M.A. conducted the final review, critical editing, and approved the manuscript for submission.

## Declaration of interests

The authors declare no conflicts of interest and confirm that they have no personal financial relationships with any of the systems or companies mentioned in this manuscript.
